# Assessing heterogeneous groundwater systems: Geostatistical interpretation of well logging data for estimating essential hydrogeological parameters

**DOI:** 10.1038/s41598-024-57435-x

**Published:** 2024-03-27

**Authors:** Musaab A. A. Mohammed, Yetzabbel G. Flores, Norbert P. Szabó, Péter Szűcs

**Affiliations:** 1 Faculty of Earth and Environmental Science and Engineering, University of Miskolc, Egyetemváros, Miskolc, 3515 Hungary; 2 College of Petroleum Geology and Minerals, University of Bahri, Khartoum, Sudan

**Keywords:** Debrecen, Pannonain basin, Factor analysis, Csókás method, Hydraulic conductivity, Environmental sciences, Hydrology

## Abstract

This research presents an unsupervised learning approach for interpreting well-log data to characterize the hydrostratigraphical units within the Quaternary aquifer system in  Debrecen area, Eastern Hungary. The study applied factor analysis (FA) to extract factor logs from spontaneous potential (SP), natural gamma ray (NGR), and resistivity (RS) logs and correlate it to the petrophysical and hydrogeological parameters of shale volume and hydraulic conductivity. This research indicated a significant exponential relationship between the shale volume and the scaled first factor derived through factor analysis. As a result, a universal FA-based equation for shale volume estimation is derived that shows a close agreement with the deterministic shale volume estimation. Furthermore, the first scaled factor is correlated to the decimal logarithm of hydraulic conductivity estimated with the Csókás method. Csókás method is modified from the Kozeny-Carman equation that continuously estimates the hydraulic conductivity. FA and Csókás method-based estimations showed high similarity with a correlation coefficient of 0.84. The use of factor analysis provided a new strategy for geophysical well-logs interpretation that bridges the gap between traditional and data-driven machine learning techniques. This approach is beneficial in characterizing heterogeneous aquifer systems for successful groundwater resource development.

## Introduction

The characterization of heterogeneous groundwater aquifers is one of the challenges that requires the integration of advanced geological and geophysical techniques to address the inherent complexities^1,2^. This spatial heterogeneity complicates the ability to accurately estimate parameters such as hydraulic conductivity and the contaminants distribution^3–6^. Traditional geological and hydrogeological methods can provide valuable insights, but they often fall short of capturing the full lithological variability within the aquifers^7–9^. On the other hand, Geophysical well logging offers a unique opportunity to attain a more comprehensive understanding of the aquifer system as it gives a continuous estimation of the aquifer characteristics^10–13^. The petrophysical and hydraulic parameters can be obtained by analyzing well-logging data using deterministic and inverse modeling^14–17^. However, formulating the mathematical problem that relates the hydrogeological parameters to the measured geophysical data is often complex and associated with a high uncertainty^18,19^.

The challenging and ill-posed nature of the hydrogeophysical inverse problem has limited the application of inversion-based models to estimate hydrogeological parameters^20,21^. One critical parameter in hydrogeology, hydraulic conductivity, presents particular difficulties due to its nonlinearity to other petrophysical and fluid properties, making its accurate prediction from geophysical data problematic^22,23^. Consequently, groundwater researchers often resort to pumping experiments^24^, which are costly and time-consuming to quantify hydraulic conductivity^25^. In this context, Csókás^26^ introduced an improved methodology for estimating hydraulic conductivity in loosely consolidated hydrogeological units, exclusively relying on well-logging data. The successful application of this method necessitates the interpretation of geophysical logs sensitive to lithology and water saturation^27^. The main advantage of well logging-based methods is their ability to give continuous profile estimation for petrophysical and hydrogeological parameters crucial to simulating and understanding the hydrodynamic conditions of the heterogeneous groundwater systems^28,29^.

Recent advancements in machine learning have opened up new possibilities for interpreting well-logging data^30–34^. Unsupervised learning methods offer efficient insights into petrophysical and hydrogeological characteristics. Among them, factor analysis, a powerful multivariate statistical approach, allows to reveal the complex interdependencies within well-logging data^35,36^ and can be used to extract details from multidimensional datasets that are not immediately observable^37^. Several works on using factor analysis for the interpretation of well logs data are reported in the literature^38,39^. For instance, Li et al.^40^ applied factor analysis for the characterization of gas-bearing formation in Sichuan Basin, China, and proved its efficiency in formation evaluation compared to the other conventional methods. A study conducted by Asfahani^41^ indicated the effectiveness of factor analysis for lithology identification. Puskarczyk^42^ identified the finer-scale variation of the lithofacies in a shale formation within the Carpathian Basin using principal component analysis (PCA). The study indicated that PCA can be successfully employed in mapping the gas-saturated and sandstone-claystone formations.

Multivariate statistical and inverse modeling approaches are mainly applied for characterizing gas and oil reservoirs. While gas and oil reservoirs have been extensively investigated, the heterogeneous groundwater aquifers present a more challenging environment due to their complex lithological nature^18^. The primary objective of this study is to use the exploratory factor analysis integrated with the Csókás method for characterization of the Quaternary aquifer in the Debrecen area. The present research stands out for its innovative interpretation of the well-logging data that bridges the gap between conventional and unsupervised learning data-driven techniques.

## Study area

### Geography

**Figure 1:**
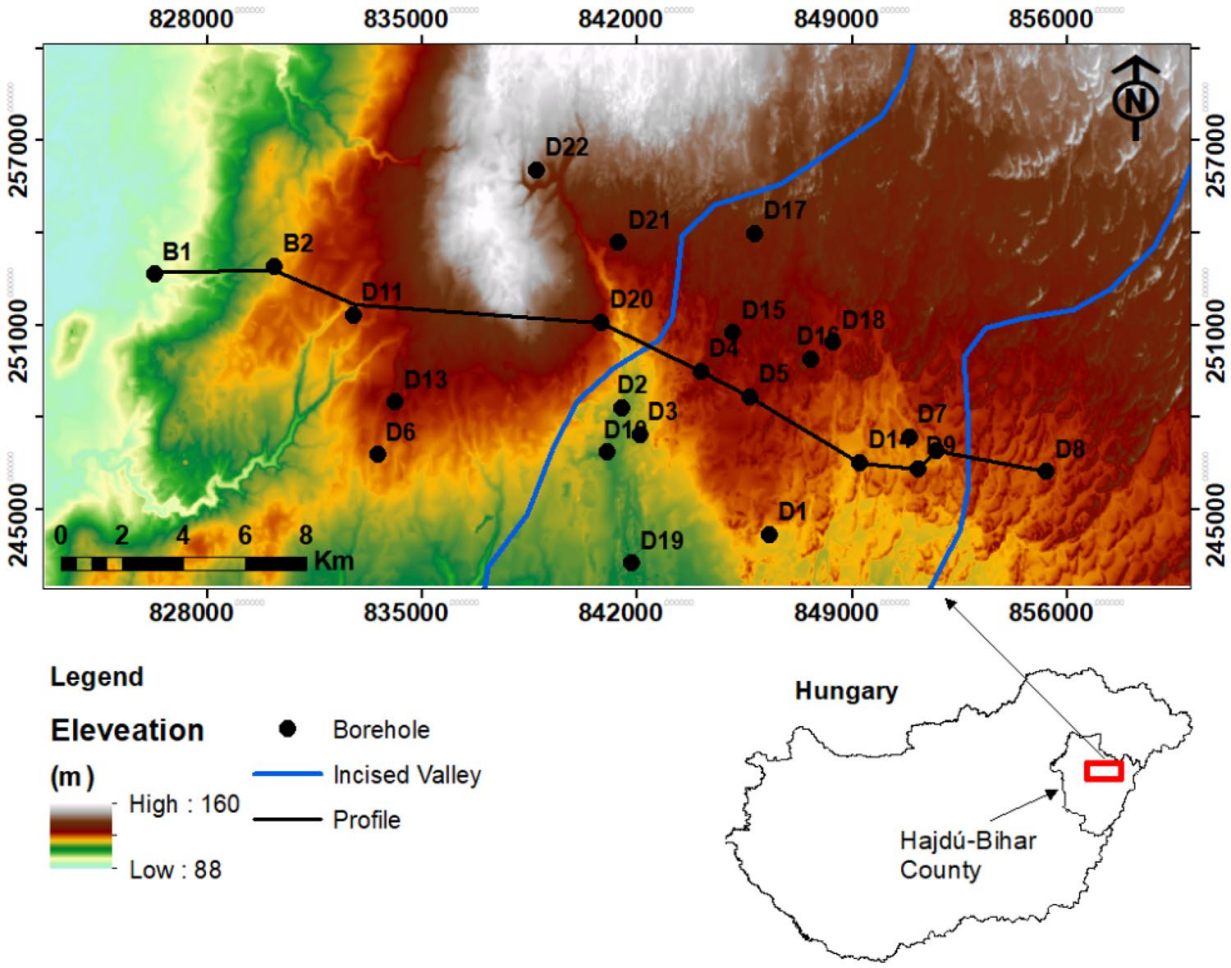
Geographic map created with ArcGIS Desktop v. 10.8^45^, showing the location of the study area within the Eastern part of Hungary.

The research site is situated around the Debrecen area, Eastern Hungary, encompassing approximately 650 km^2^ (Fig. 1). It is integral to the Great Hungarian Plain (GHP) in which substantial variations in land elevation have transpired due to contemporary tectonic movements, erosion, and extensive sedimentation processes^43^. The geological movements have notably influenced the topography in the study area, leading to an elevation ranging from 88 to 160 m above sea level (a.s.l). The region’s climate can be characterized as predominantly continental, with annual mean temperatures ranging from 10° to 11 °C. The annual precipitation varies from 550 to 600 mm, and potential evapotranspiration ranges between 600 and 700 mm/year^44^.

### Geology

**Figure 2:**
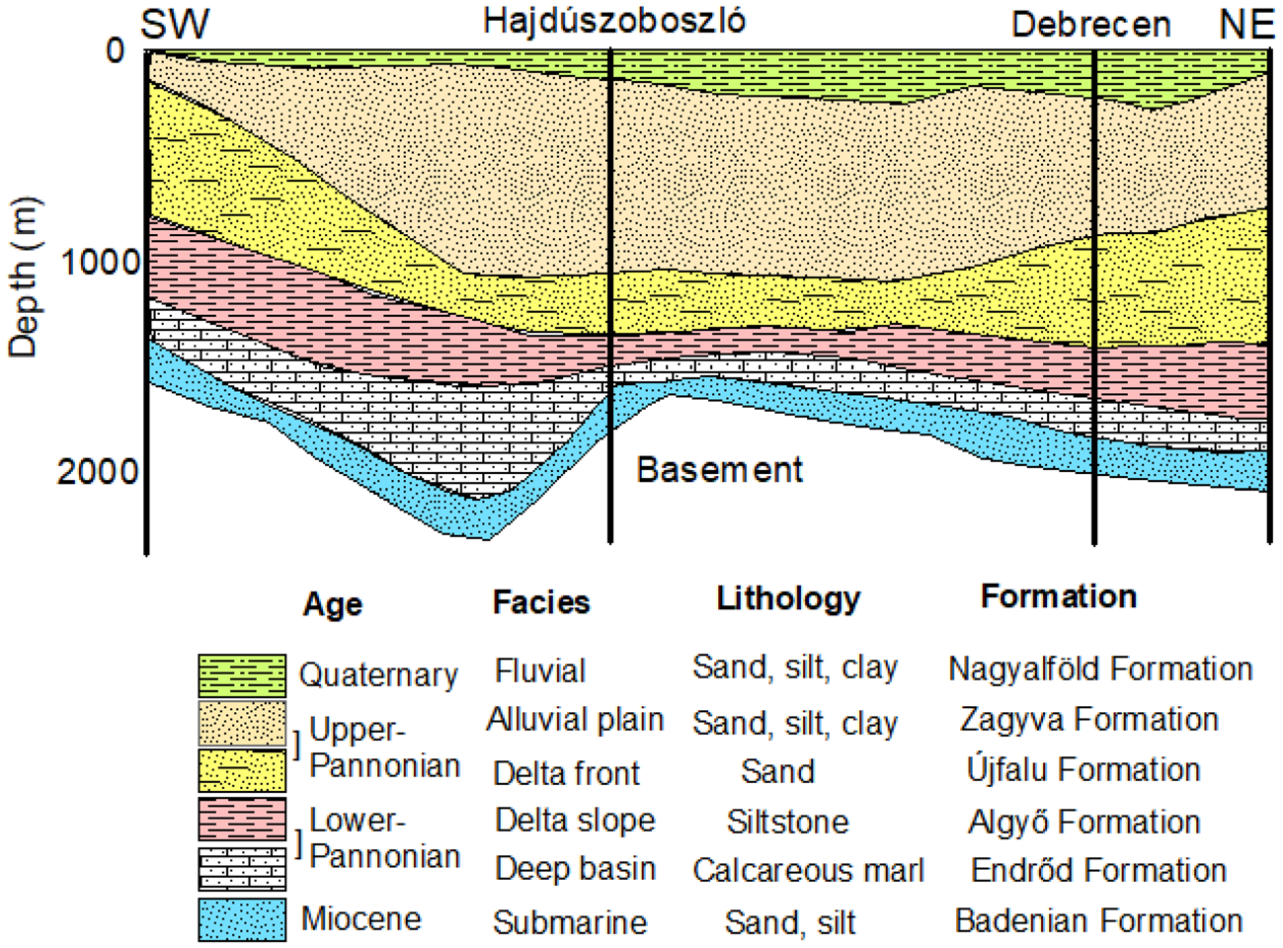
Geological cross-section showing the main lithological formations in the study area modified after Juhász^52^ and Tóth and Almási^49^.

The research consists of diverse geological formations including Mesozoic basement rocks, Miocene deposits, Pannonian layers, and Quaternary Formation (Fig. 2). The Mesozoic rocks are composed of metamorphic and igneous rocks, and they are primarily associated with the Tisza Mega-Unit^46,47^. These rocks encompass a variety of rock types, such as granites, gabbros, and basalts, alongside schists and phyllites^48^. The Miocene Formation is characterized by an assortment of sedimentary rocks, encompassing marl, sandstones, and claystone^49^. The Pannonian sediments are classified into two distinct parts, namely the Lower and Upper Pannonian^47^. During the early stages of the Lower Pannonian period, the initially deposited coarse-grained sandstone and coastal sandy conglomerates underwent lateral transformations into siltstone, known as the Algyő Formation. Simultaneously, there was the development of calcareous marl and limestone, referred to as the Endrőd Formation^49^. Conversely, the Upper Pannonian era comprises a succession of sedimentary layers, encompassing sandy delta plain and delta front sediments, interspersed with alluvial siltstone, sandstone, clay, marl, and quartz pebbles. These particular deposits are observed within the Újfalu and Zagyva formations^50^. The surface of the GHP predominantly consists of Quaternary deposits. These deposits encompass fluvial sediments, river sediments, and sandy loess. The thickness of Quaternary deposits in the research area varies from 80 to 150 m. These deposits are categorized into three segments: upper, middle, and lower Pleistocene beds^47^. The lower and upper sections predominantly comprise river and overbank sediments, while the middle section predominantly encompasses coarse-grained fluviolacustrine sediments^51^.

### Hydrogeology

In the Great Hungarian Plain (GHP), five hydrostratigraphic units were identified based on their lithology and chronostratigraphy. These units are the Pre-Neogene impermeable layer, the Pre-Pannonian aquifer, the Endrőd confining layer, the Algyő confining layer, and the Nagyalföld water-bearing stratum^49^. The Nagyalföld Aquifer, which encompasses the Újfalu and Zagyva Formations along with Quaternary sediments, has been recognized as the main aquifer with a permeability exceeding 1000 mD^49,53^.

**Figure 3:**
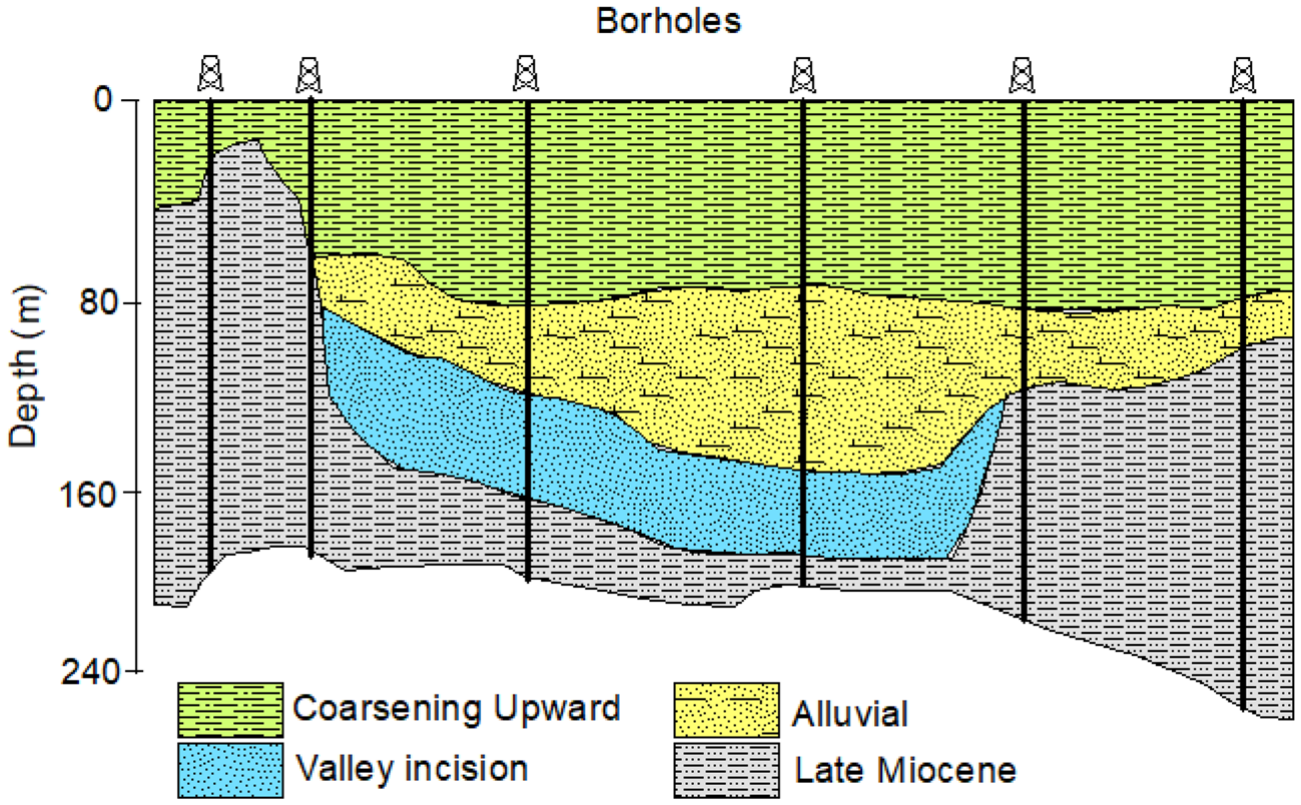
The main hydrostratigraphical units within the Nagyalföld aquifer, modified after Flores et al.^44^.

Recently, Flores et al.^44^ conducted an extensive regional-level hydrostratigraphical investigation, concentrating on the upper section of the Nagyalföld aquifer. Their findings revealed that the key hydrostratigraphic components in their study encompass the Pre-Quaternary and Quaternary sequences (Fig. 3). The Pre-Quaternary sequence of the Late Miocene is distinguished by substantial layers of silt with occasional intercalated fine sand. In contrast, the Quaternary sequence is characterized by three hydrostratigraphic divisions, ordered from older to younger. The first is an incised valley unit, described as an elongated body of sand and gravel with minimal clay content. Above it, the alluvial unit is depicted as a succession of three consecutive sand bodies with significant horizontal variability and deposits of silty clay. Finally, the coarsening upward unit is described as a sequence displaying pronounced heterogeneity, featuring clay, silt, and sand bodies. The observations have unveiled the existence of two distinct hydraulic systems in the study area. In the upper system, groundwater flow is predominantly governed by gravitational forces, while the lower system experiences overpressure^10^. Hydraulic interaction between these two systems frequently occurs, particularly in areas where low-permeability layers exert outward pressure^52^.

## Materials and methods

This study used geophysical well logging data to identify and characterize groundwater aquifers in the Eastern Hungary region surrounding Debrecen. In this work, the aquifer geometry and the petrophysical and hydrogeological parameters of the Quaternary aquifers in the study region are defined utilizing data collected from twenty-four (24) boreholes. This study employed three well logs including spontaneous potential (SP), natural gamma ray (NGR), and deep normal resistivity (RS), and analyzed using Csókás method and factor analysis.

### Csókás approach

Csókás^26^ model is used for estimating hydraulic conductivity from the well logs data. This method can be seen as an empirically refined version of the equations proposed by Kozeny^54^ and Carman^55^. The Kozeny-Carman equation takes into account several key parameters, such as the density of water (ρ_w_), viscosity (μ), porosity (φ), the dominant grain size of the aquifer materials (d), and the acceleration due to gravity (g). The Kozeny-Carman-based hydraulic conductivity (K_KC_) can be estimated using Eq. (1).

1 $${K}_{KC}= \frac{{\rho }_{w}\mathcal{g}}{\mu } \frac{{d}^{2}}{180} \frac{{\varphi }^{3}}{{(1- \varphi )}^{2}}.$$

Csókás approach proves to be particularly applicable in situations involving lossy geological formations. This suitability is established through an empirical connection between the effective grain size of water-saturated sediments (d_10_) and the formation factor (F = $\frac{{R}_{0}}{{R}_{w}}$) (Eq. 2). Alger^56^ investigation revealed that, apart from the porosity ($\varphi$), the resistivity of water (${R}_{w}$) also exerts an influence on the formation factor. In this research, the effective porosity is estimated using Eq. (3)^57^, considering the shale volume (${V}_{sh}$) present in the geological formation. The shale volume however, is estimated using Larianov^58^ equation (Eqs. 4 and 5). Consequently, the hydraulic conductivity (K, m/s), calculated using the Csókás method can be determined using Eq. (6).

2 $${d}_{10}= {C}_{d} log F,$$

3 $${\varphi }_{e}=\varphi \times \left(1- {V}_{sh}\right),$$

4 $${V}_{sh}=0.33 \left({2}^{2*{I}_{\gamma }}- 1\right),$$

5 $${I}_{\gamma }= \frac{{GR}_{log}- {GR}_{min}}{{GR}_{max}- {GR}_{min}},$$

6 $$K= {C}_{k }\frac{\varphi }{{\left(1- \varphi \right)}^{4}} \frac{{\left(log \frac{{R}_{0}}{{R}_{w}}\right)}^{2}}{{\left( \frac{{R}_{0}}{{R}_{w}}\varphi \right)}^{1.2}},$$

where ${\varphi }_{e}$ is the effective porosity, ${I}_{\gamma }$ is the gamma-ray intensity, which is calculated using a linear formula that uses the gamma-ray response of the log ${(GR}_{{\text{log}}})$, minimum ${(GR}_{{\text{min}}})$ and the maximum ${(GR}_{max})$ gamma-ray. C_k_ is the proportionality constant and has the value 855.7 * 5.22 * 10^–4^.

### Exploratory factor analysis (FA)

Factor analysis is an unsupervised machine learning method that facilitates the reduction of complex datasets into a more manageable set of factors. In this study, factor analysis was employed to extract factor logs representing the largest portions of variance within the dataset from the analysis of the available well logs of SP, NGR, and RS^59^. These factor logs are then linked to shale volume estimated using the Larionov^58^ equation and hydraulic conductivity determined by the Csókás^26^ method. The correlation of factor logs with these parameters aids in developing site-specific equations that facilitate direct connections between the factor log and aquifer parameters that can be used as alternatives to the existing methods.

During the initial stages, standardization of well logs was necessary, given the use of different probes and, consequently, varying measurement units (Eq. 7), followed by the integration of data into a matrix (D) (Eq. 8), and the application of a factor analysis model (Eq. 9).

7 $$\check{D}_{il} = \frac{{\left( {D_{il} - \overline{D}_{l} } \right)}}{{\sqrt {\frac{1}{N} \mathop \sum \nolimits_{i = 1}^{N} \left( {D_{il} - \overline{D}_{l} } \right)^{2} } }},$$

8 $$D=\left[\begin{array}{ccc}{SP}_{1}& {GR}_{1}& {RS}_{ 1}\\ {SP}_{2}& {GR}_{2}& {RS}_{2}\\ \vdots & \vdots & \vdots \\ {SP}_{i}& {GR}_{i}& {RS}_{i}\\ \vdots & \vdots & \vdots \\ {SP}_{n}& {GR}_{n}& {RS}_{n}\end{array}\right],$$

9 $$\boldsymbol{\hat{D} }=F{W}^{T}+E.$$

In this context, ${\boldsymbol{\hat{D} }}_{{\varvec{i}}{\varvec{l}}}$ represents the scaled data for the n-th observation within the l-th well log. $\overline{D}_{l}$ corresponds to the average value of the unprocessed data from the l-th well log, where L is the total number of borehole geophysical tools, and N is the count of measuring points in the specified depth range. F is the factor score matrix of dimensions N by M, where M is the number of extracted factors, W is the factor loading matrix of dimensions L by M. E is the matrix of residuals with dimensions N by L, and T represents the matrix transpose operator.

The primary factor explains the majority of the variation in the dataset, while the subsequent factors contribute to a relatively smaller portion of the variance. The factor loading matrix, which measures the degree of association between the factors and the actual data, offers precise weights for each data category. Because the factors are statistically uncorrelated, the correlation matrix of the observed data can be indicated using Eq. (10) as

10 $$R= \frac{1}{N} {D}^{T}D=L{L}^{T}+\Psi .$$

In this context, Ψ represents a diagonal matrix containing specific variances. When Ψ takes on a value of 0, the issue can be resolved through the solution of an eigenvalue problem. If Ψ differs from 0, the factor scores are determined using the maximum likelihood method, and the subsequent objective function is then optimized to simultaneously estimate both L and Ψ^60^ (Eq. 11).

11 $$\Omega \left(L,\Psi \right)=tr {(R,L{L}^{T}-\Psi )}^{2}=min.$$

Factor loadings are usually subjected to an orthogonal transformation to enhance the interpretability of factors, as proposed by Ref.^37^. In this study, factor rotation was carried out using the varimax technique, following Kaiser^61^ approach. Factor scores can be derived by applying a linear approach with the assumption of linearity^62^ (Eq. 12).

12 $${F}^{T}={({L}^{T}{\Psi }^{-1}L)}^{-1}{L}^{T}{\Psi }^{-1}{D}^{T}.$$

The Pearson^63^ (R) and Spearman^64^ (ρ) correlation coefficients are utilized to assess the relationships between the extracted factor logs, well logs, and petrophysical and hydrogeological parameters. Pearson correlation coefficient evaluates the strength and direction of the linear relationship between the continuous variables while Spearman rank correlation coefficient measures the strength and direction of the monotonic relationship. Both coefficients range from − 1 to 1, with 1 indicating a perfect positive relationship, 0 indicating no relationship, and − 1 indicating a perfect negative relationship. These coefficients provided simple sensitivity analysis to evaluate the associations between well logs and the extracted factor logs.

## Results

**Figure 4:**
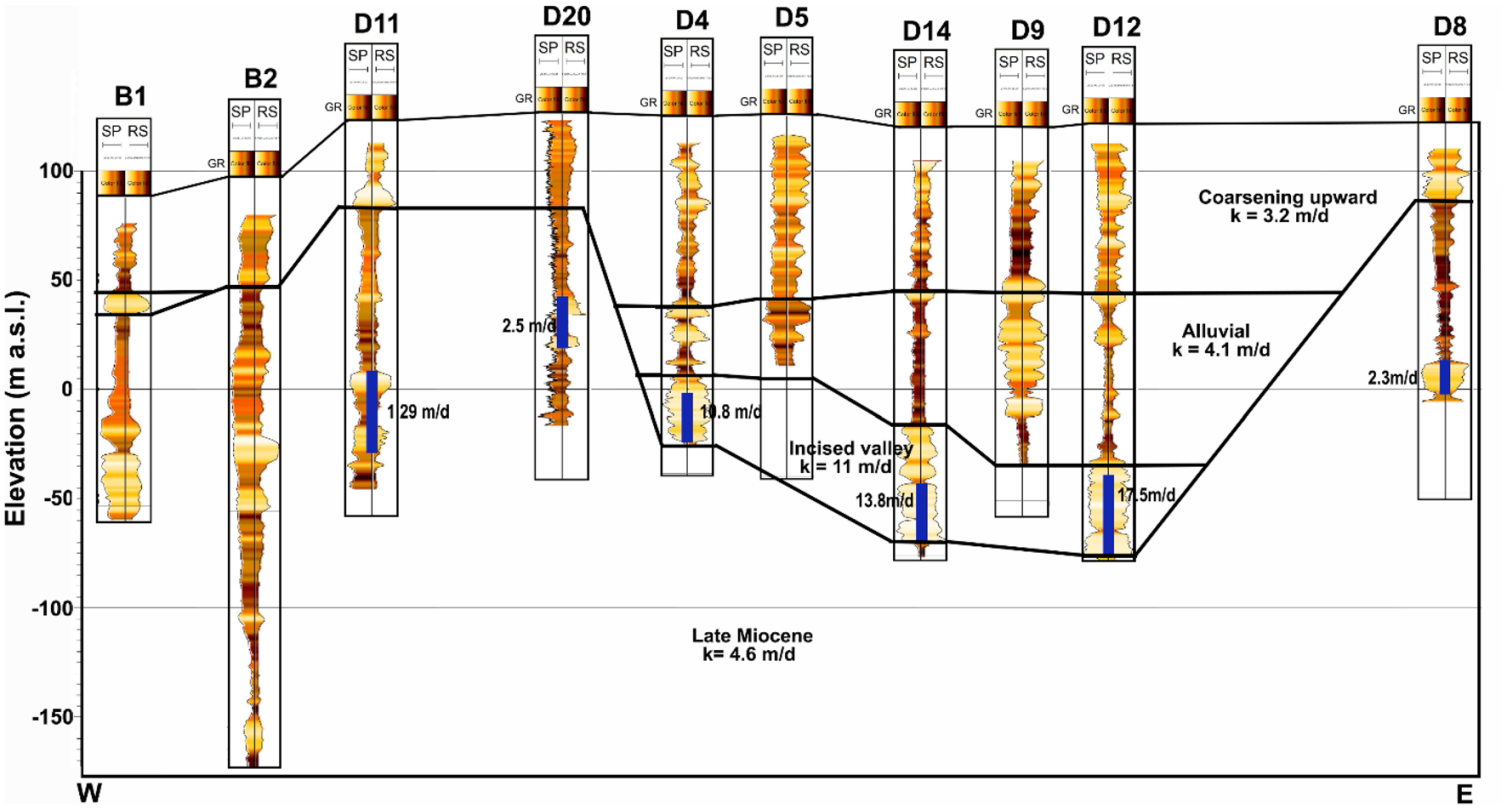
2D geophysical profile showing the pattern of the SP, GR, and SP logs and the distribution of the main hydrostratigraphical units in the study area.

This research introduces factor analysis for the interpretation of well logs for estimation of shale volume (V_sh_), effective porosity (${\varphi }_{e}$), and hydraulic conductivity (K) of the Quaternary aquifers in the Debrecen area. The data is analyzed in 1D along the boreholes, and the obtained results are interpolated in 2D along a profile. The distribution of the borehole along the profile is illustrated in Fig. 4, with the stratigraphic bounding surfaces described by Flores et al.^44^. These surfaces are created with the geometrical convergence interpolation^65^ of the identified well tops following the sequence stratigraphical principles^66^. The hydrostratigraphic units in the area from the bottom to top are the Late Miocene, incised valley, alluvial, and coarsening upward units (Fig. 4). The Late Miocene unit is characterized by low occurrence of silty sand lithologies embedded in thick silty clay sequences while the incised valley unit is dominated by a thick sequence of gravel and sand deposits. Over them, the alluvial unit is characterized by the occurrence of two sandy channel deposits, embedded into a thick clayed floodplain deposit. The coarsening upward unit is characterized by coarsening upward facies that are made up of a successive intercalation between clay, silt, and sand. Several aquifer units are developed within these hydrostratigraphical units with the incised valley deposits hosting the main aquifer in the study area^51^.

### FA-based shale volume

The well logging data comprises a total of 34,328 data points along 24 boreholes and is divided into two parts in which 60% of the data is used for correlation and 40% of the data for testing the resulting relationship. The first factor explained 81.7% of the total variance, indicating its robust representation of underlying features in the dataset. A higher positive loading is given to NGR (0.70) and medium negative loading to RS (− 0.57).

**Figure 5:**
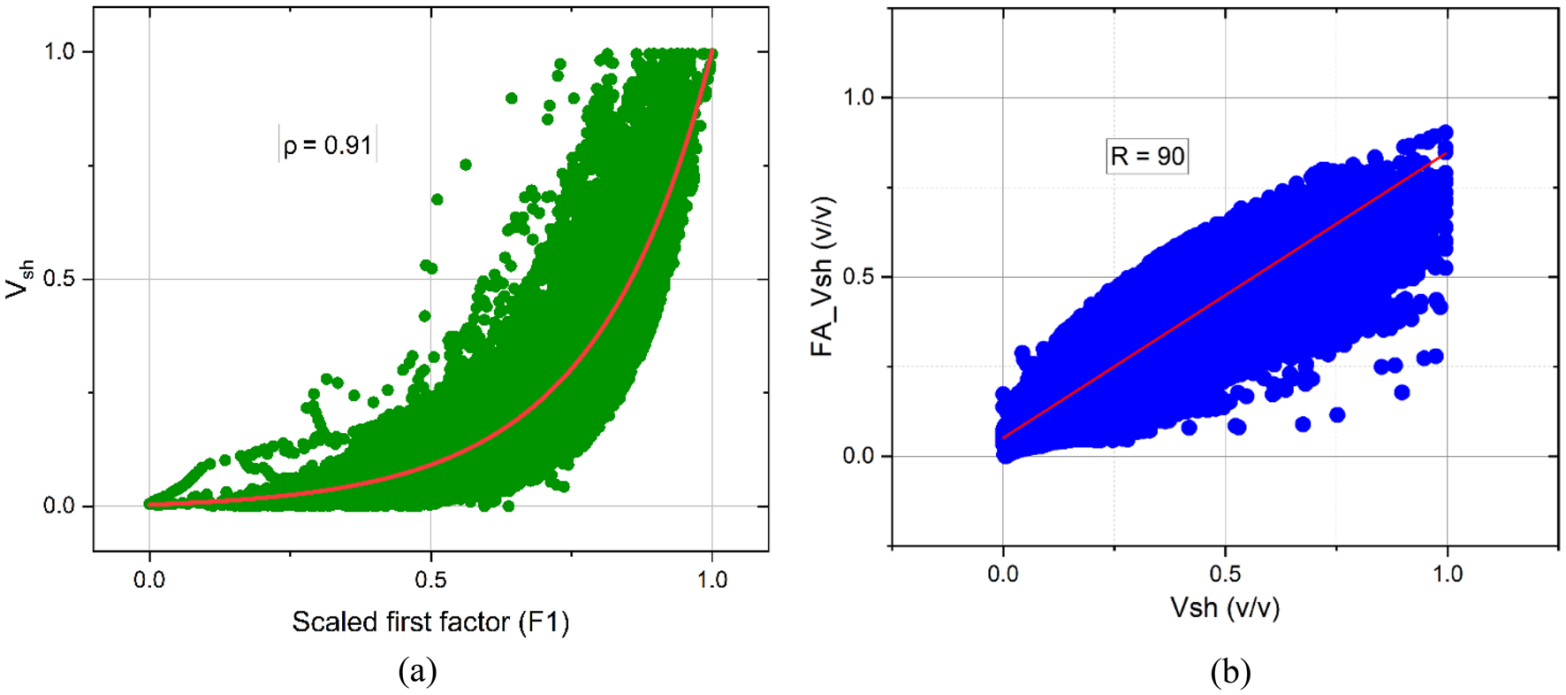
(**a**) The relationship between the scaled first factor and shale volume and (**b**) the correlation between the factor analysis-based and Larionov shale volume.

The scores of the first factor of the 60% of the data are correlated to shale volume estimated from the Larionov equation and yielded a strong exponential relationship with a Spearman correlation coefficient of 0.91 (Fig. 5a). This relationship underscores the importance of the first factor as a powerful proxy for shale volume^59^. Accordingly, a site-specific equation is obtained that linked shale volume (V_sh_) to the scaled first factor (F_1_) and written as

$${V}_{sh}=a{e}^{b{F}_{1}},$$

where a and b are site-specific constants from the local regression that is given with 95% confidence. The average values of a = 0.0153 [0.0067, 0.281] and b = 4.2276 [3.736, 5.2244]. To evaluate the practical utility of the relationship between the first factor and shale volume, 40% of the data is used. Accordingly, the correlation between shale volume obtained from factor analysis and Larionov^58^ method is illustrated in Fig. 5b. The promising results obtained from this validation process, where the correlation coefficient reached 0.90, underscores the applicability of the factor analysis-based shale volume estimation.

**Figure 6:**
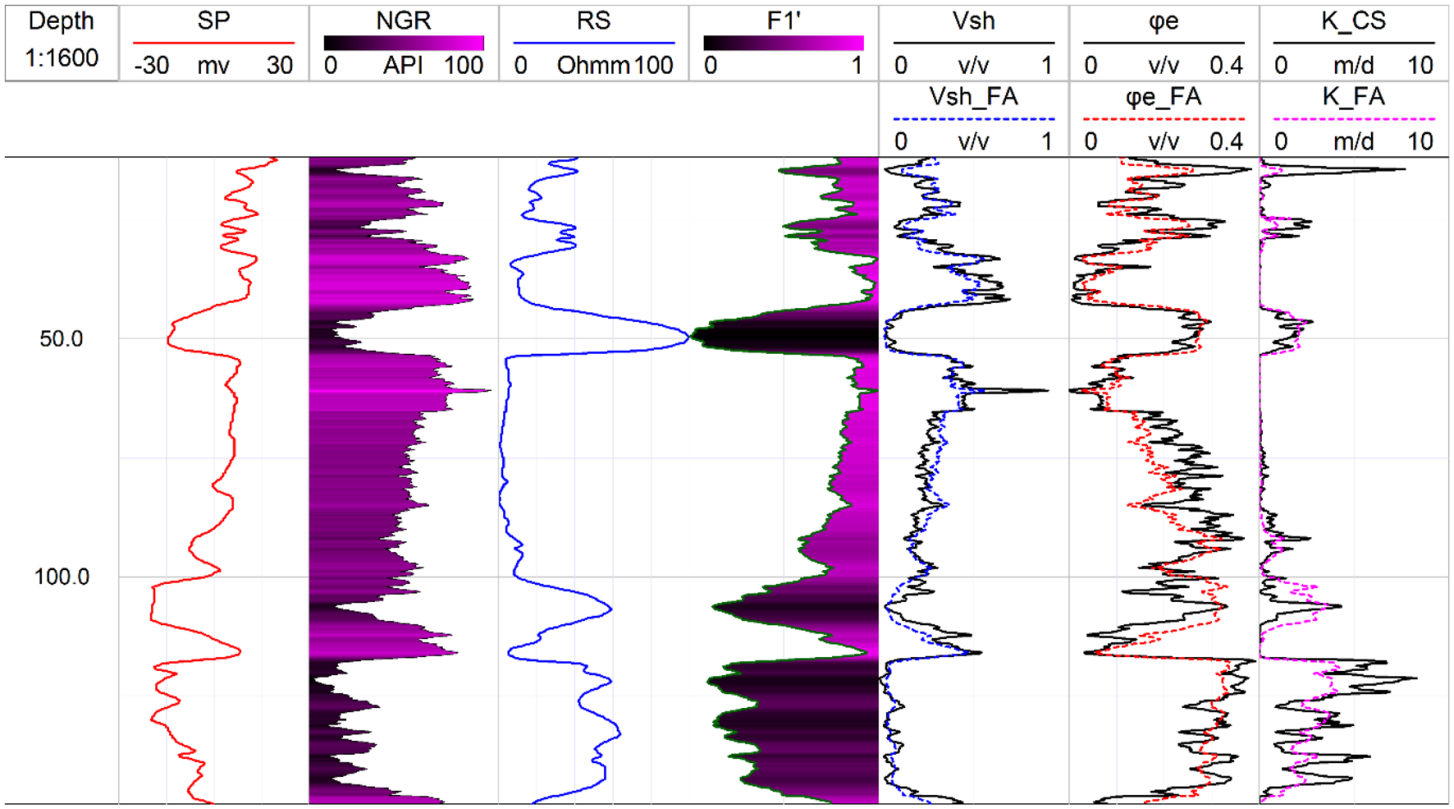
1D analysis and interpretation of the well logs data in borehole B1 showing the estimated petrophysical and hydrogeological using conventional and factor analysis methods.

**Figure 7:**
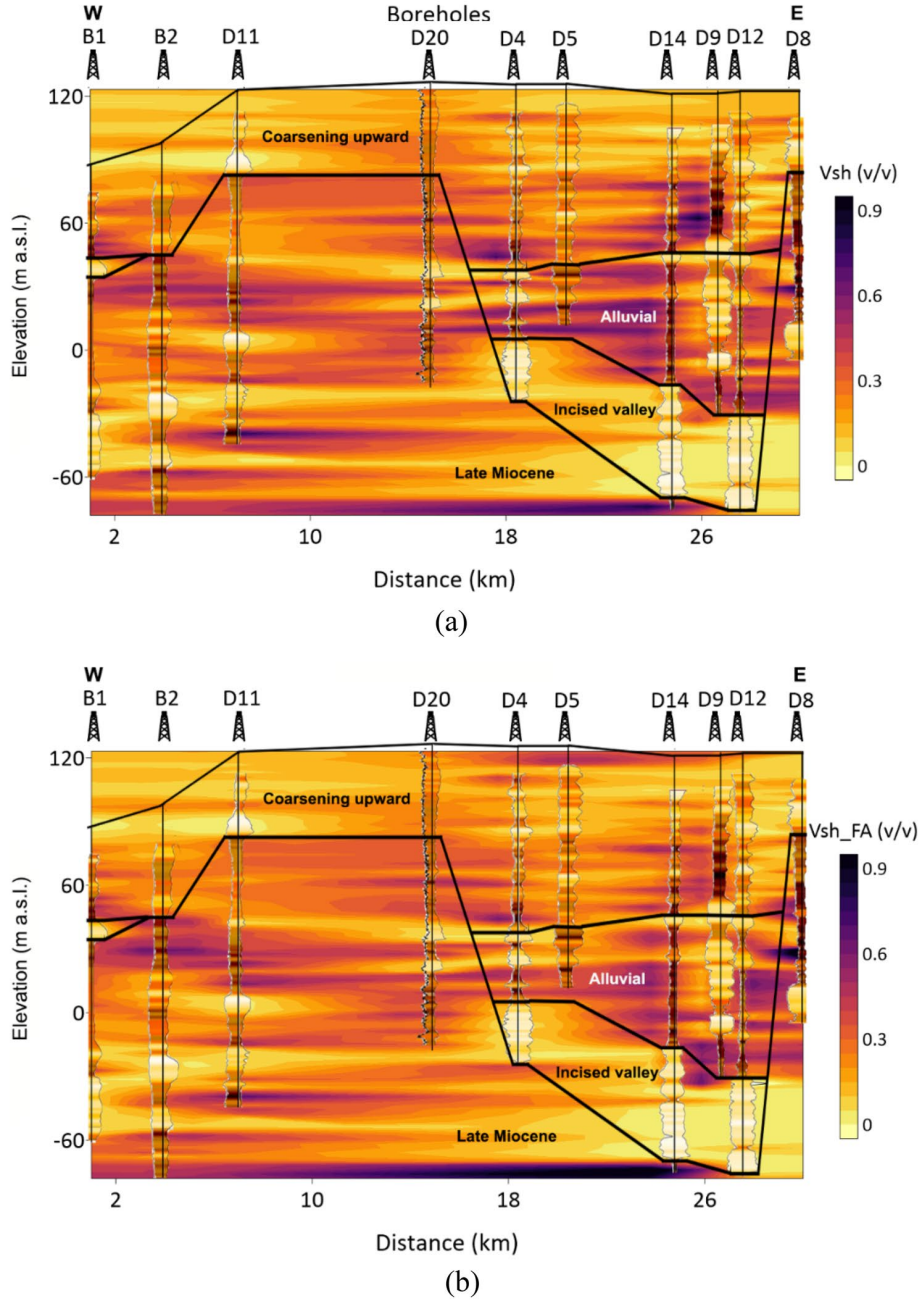
The estimated shale volume along the profile based on the (**a**) Larionov equation and (**b**) factor analysis.

**Figure 8:**
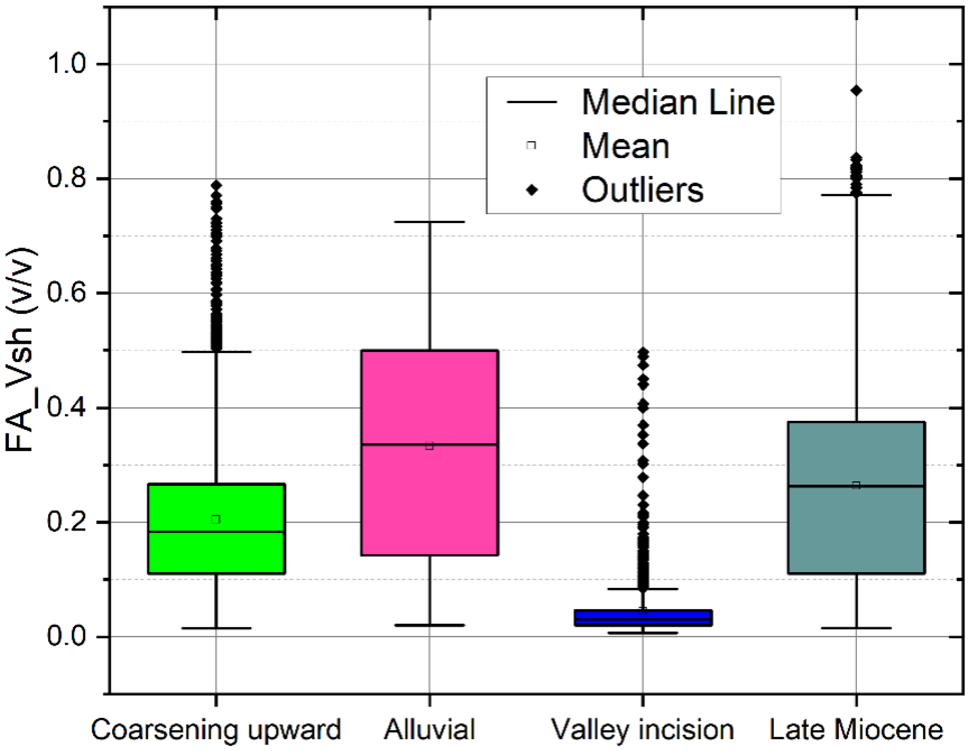
Box plot showing the statistical summary of the shale volume for the main hydrostratigraphical unit.

**Figure 9:**
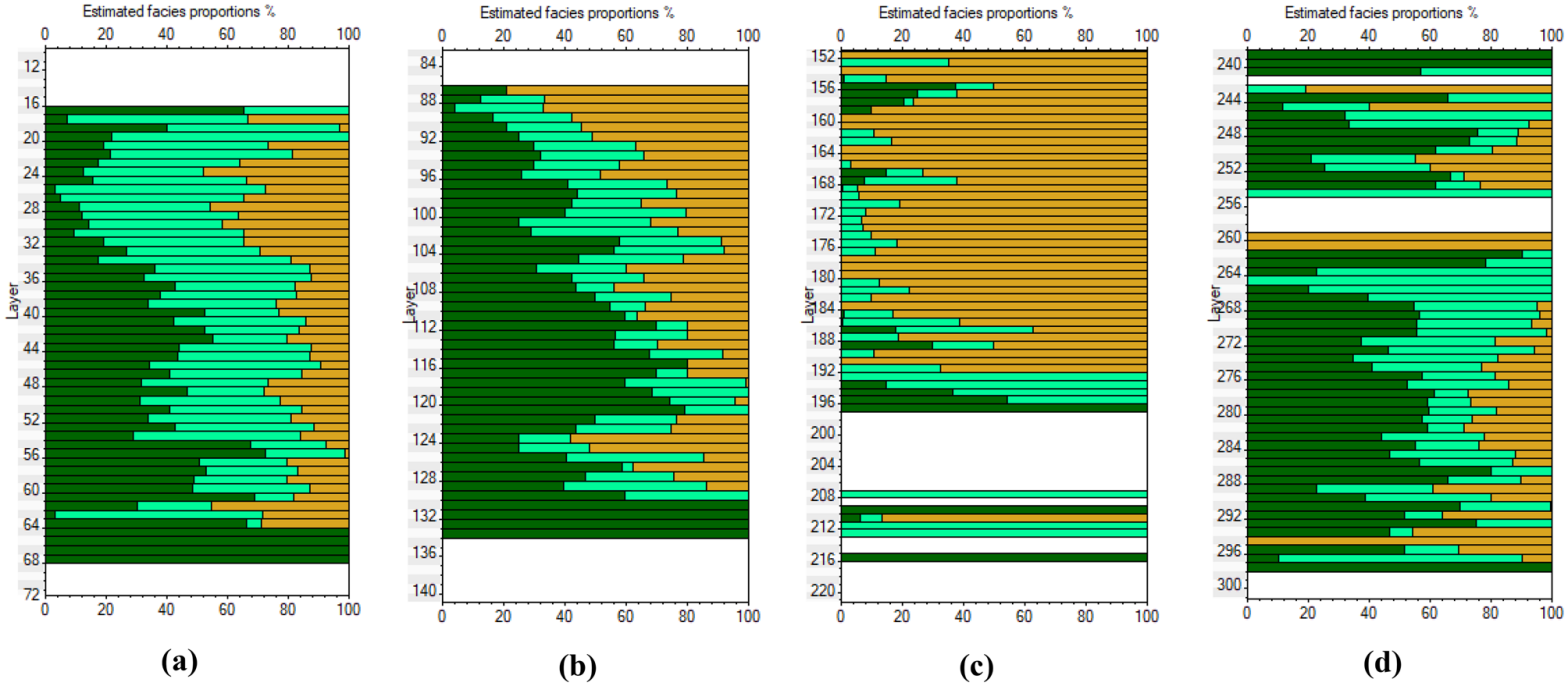
The estimated facies proportion for (**a**) coarsening upward, (**b**) alluvial unit, (**c**) valley incision, and (**d**) Late Miocene unit.

Based on the obtained relationships, the FA-based shale volume is estimated in 1D (Fig. 6) and 2D along the profile (Fig. 7). The 2D spatial variation of the Larionov^58^ equation-based shale volume (Fig. 7a) is compared to FA-shale volume (Fig. 7b). The comparison between the two approaches showed a close agreement. The descriptive statistics of the FA-based shale volume are illustrated in Fig. 8. The shale volume values are then compared to lithofacies proportion calculations based on the analysis of the well-logs data assuming 2 m layer thickness (Fig. 9). The FA-based shale volume of the coarsening upward unit exhibited significant variability, ranging from 0.05 to 50%, with a mean value of 20%. The lithofacies proportion (Fig. 9a) indicated that this unit consists of 37.7% clay, 42.5% silt, and 19.8% sand. The alluvial unit displayed almost similar variability in shale volume, ranging from 0.07 to 72%, with a mean of 34%. This unit consists of 41.9% clay, 26.9% silt, and 3.2% sand (Fig. 9b). The valley incision unit exhibited a relatively uniform distribution of shale volume, varying from almost zero (0.5%) to 9%. Consequently, the facies analysis indicated that this unit is composed of 78.7% sand (Fig. 9c). The Late Miocene unit displayed shale volume variations from 0.05 to 77%, with a mean value of 26%. This unit is dominated by clay and silt layers that make up more than 80% of the unit (Fig. 9d).

### Effective porosity

**Figure 10:**
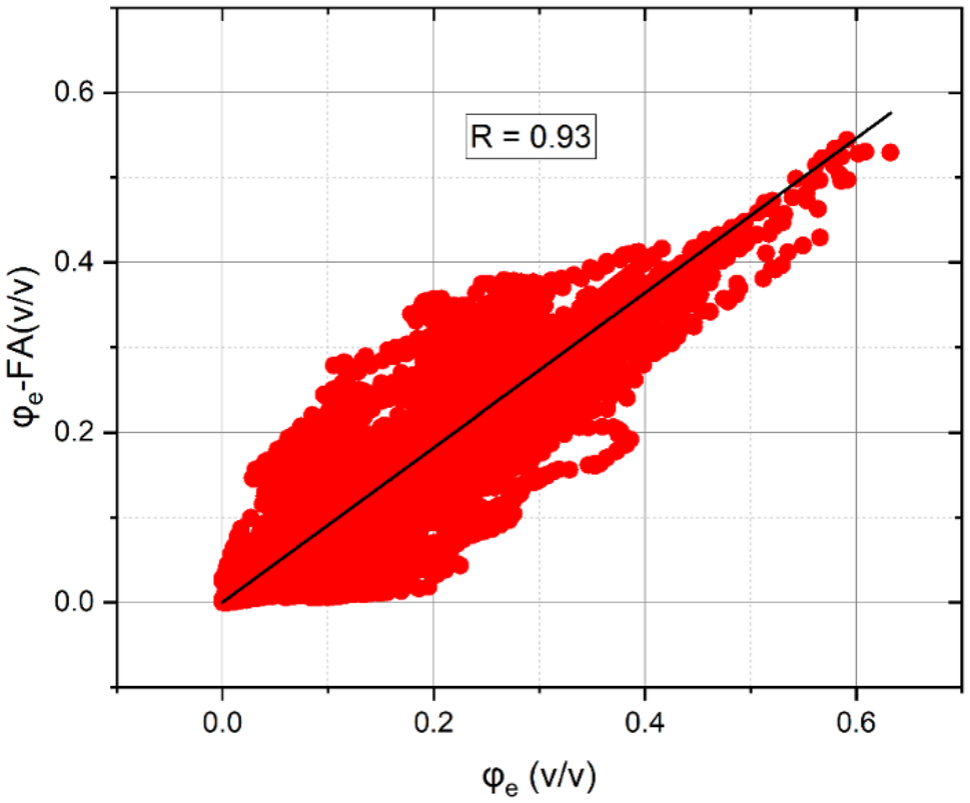
The correlation between the effective porosity using conventional methods and factor analysis.

**Figure 11:**
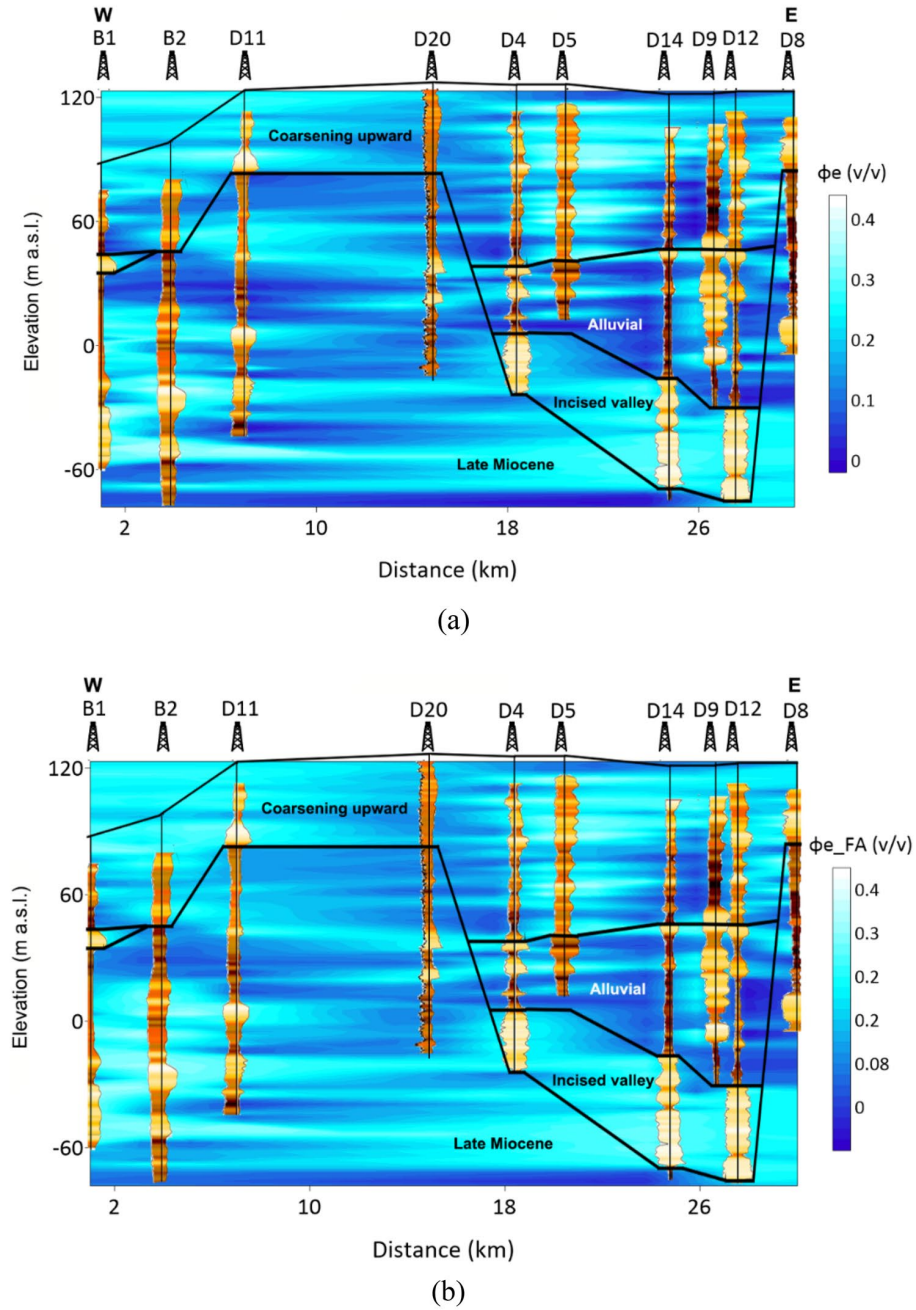
The estimated effective porosity along the profile based on the (**a**) empirical method and (**b**) factor analysis.

**Figure 12:**
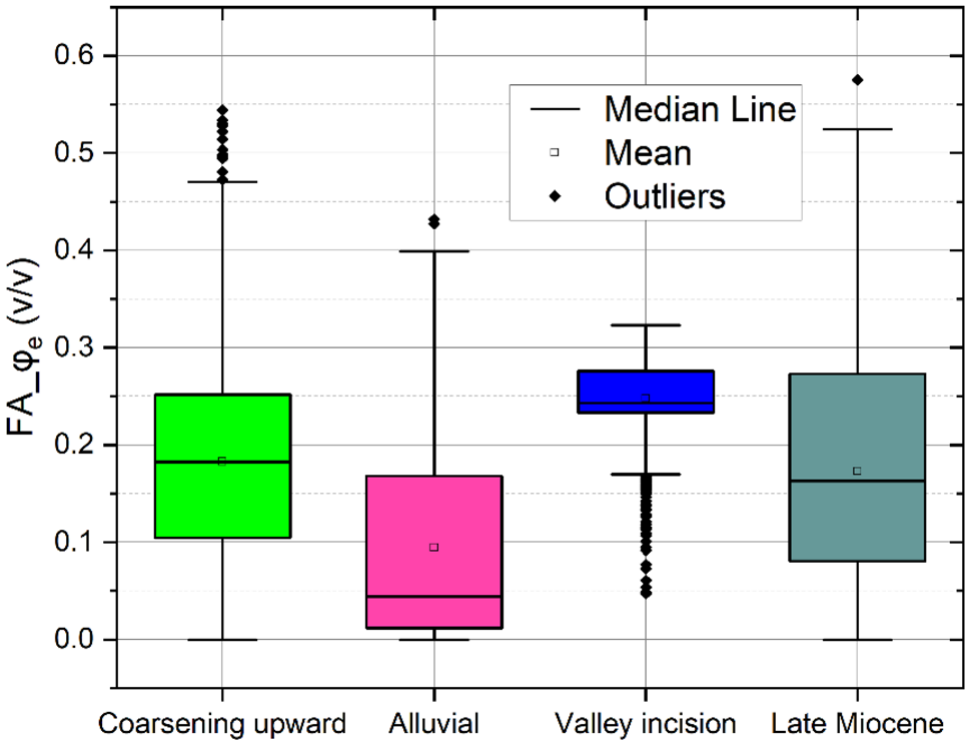
Box plot showing the statistical summary of the calculated effective porosity for the main hydrostratigraphical unit.

The effective porosity is essential for assessing the rate of groundwater flow within the aquifer^67^. In this study, the FA-based shale volume is substituted into Schlumberger^57^ formula for a more practical estimation of effective porosity. The obtained parameters from the FA approach are compared to those of conventional approaches and showed a close agreement with a 0.93 correlation coefficient (Fig. 10). Figure 11 shows the 2D interpolation of the effective porosity obtained from the empirical method (Fig. 11a) and factor analysis (Fig. 11b), in which a close agreement between the two approaches is indicated. As a result, the obtained effective porosity for the hydrostratigraphical units is illustrated using a Box plot (Fig. 12). The effective porosity of the coarsening upward unit exhibited notable variability, ranging from nearly impermeable conditions at 0.005% to highly permeable conditions at 47%, with an average of 18%. The effective porosity of the alluvial unit displayed a similar pattern ranging from 0.004 to 44%. The valley incision unit demonstrated a more uniform distribution of effective porosity, varying from 16 to 33%, with a mean of 25% while the Late Miocene unit exhibited effective porosity values ranging from almost zero to 50%, with a mean of 16%.

### Hydraulic conductivity

**Figure 13:**
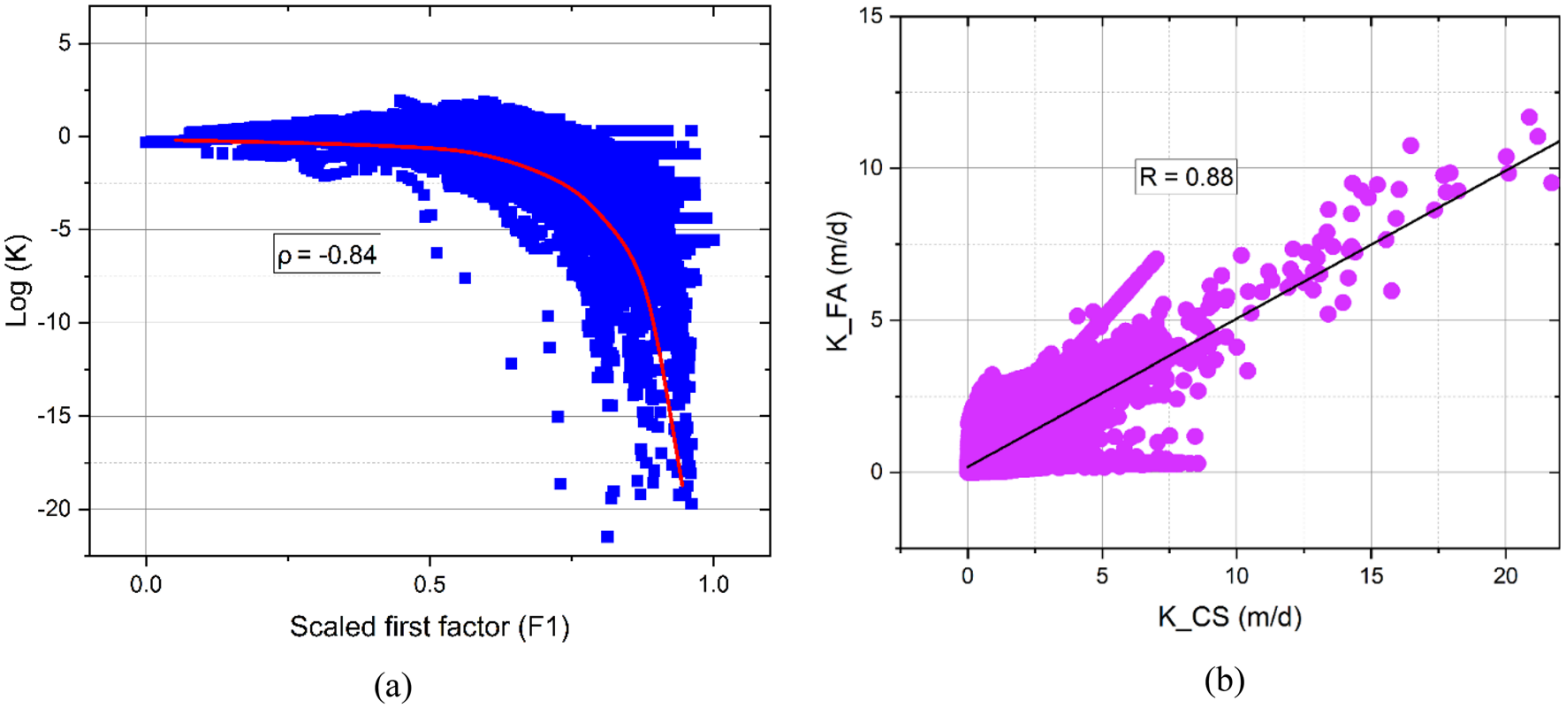
(**a**) The relationship between the scaled first factor and hydraulic conductivity and (**b**) the correlation between the factor analysis-based and Csókás method shale hydraulic conductivity.

**Figure 14:**
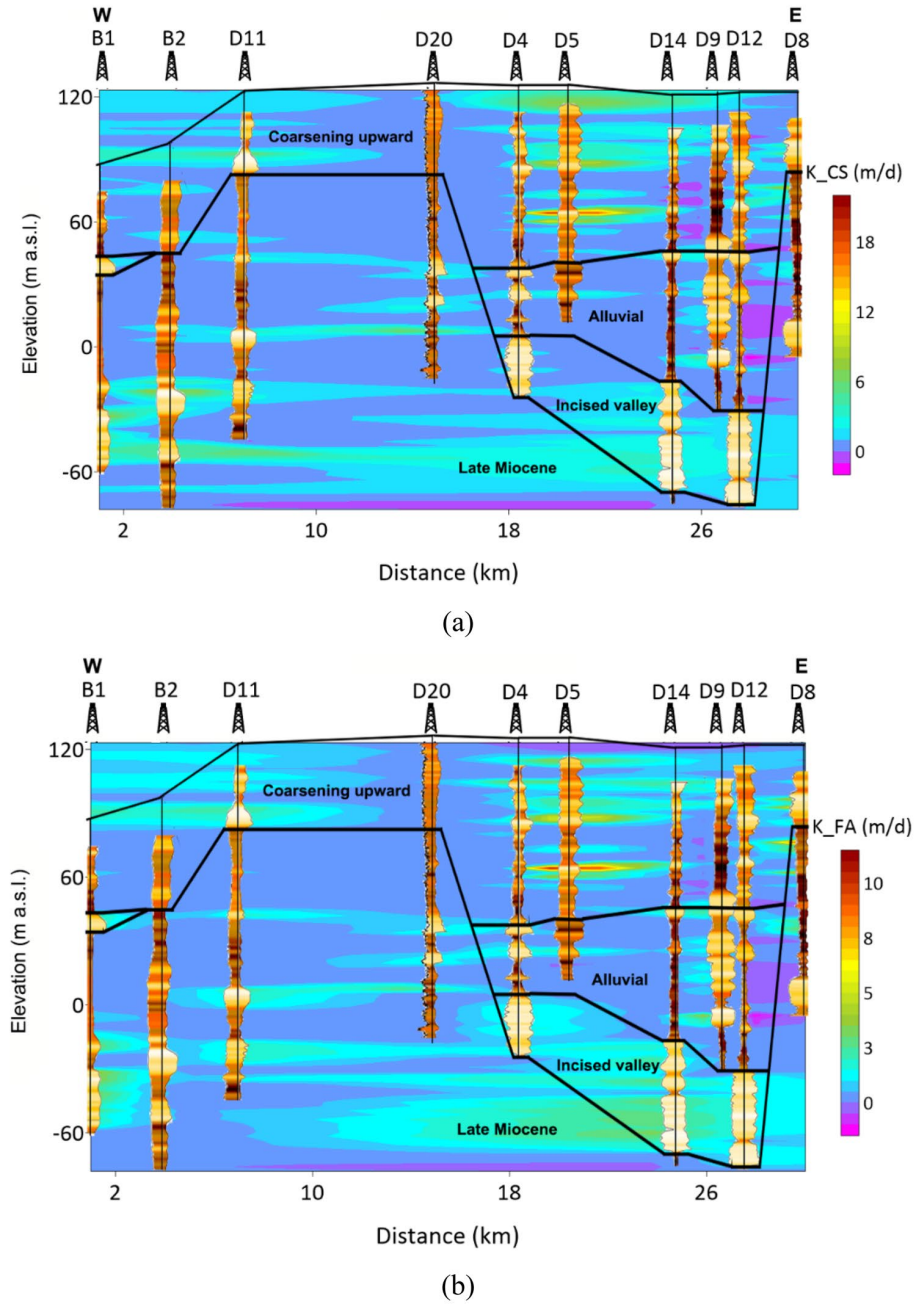
The calculated hydraulic conductivity along the profile based on the (**a**) Csókás method and (**b**) factor analysis.

**Figure 15:**
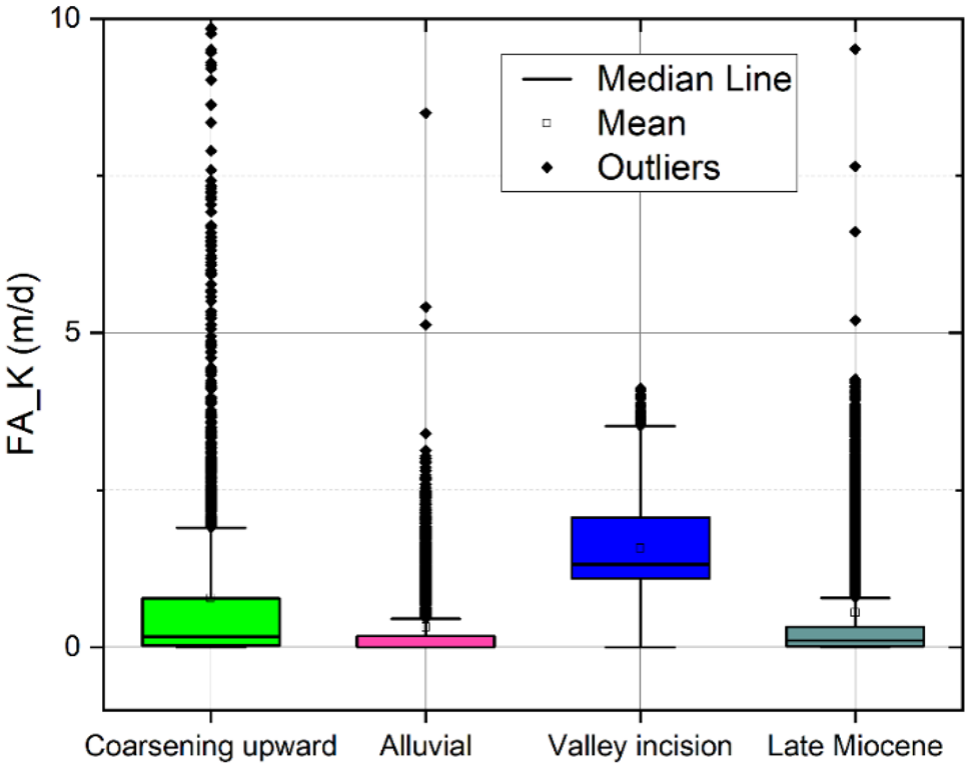
Box plot showing the statistical summary of the FA-based hydraulic conductivity for the main hydrostratigraphical unit.

In the sedimentary clastic formations, the hydraulic conductivity and the amount of shale are generally inversely correlated^68^. In this research, the hydraulic conductivity values obtained from the Csókás method are correlated to the first factor. Accordingly, a strong negative nonlinear relationship with a correlation coefficient of − 0.84 is detected^69^ (Fig. 13a) that takes the following form,

$${\text{log}}\left(K\right)=a{\left(1-{F}_{1}^{b}\right)}^{c}+d,$$

where a, b, c, and d represent site-specific regression coefficients. These coefficients showed values of 19.2, 4.27, 0.2, and – 19, respectively. The correlation between the hydraulic conductivity of the factor analysis and the Csókás method is shown in Fig. 13b, in which a close agreement (R = 0.88) is indicated. Accordingly, the hydraulic conductivity is mapped into 2D to reveal the vertical and horizontal variation (Fig. 14). The descriptive statistics of the FA-based hydraulic conductivity are illustrated in Fig. 15. The hydraulic conductivity of the coarsening upward unit ranged from nearly impermeable conditions at 0.005 m/d to more conductive zones with values of up to 2.3 m/d. The mean hydraulic conductivity for this unit was approximately 0.5 m/d. In the alluvial unit, it varied from 0.004 to 0.7 m/d. Notably, the valley incision unit demonstrated a more uniform distribution of hydraulic conductivity, with values ranging from 0.05 to 3.6 m/d and a mean value of approximately 1.3 m/d. The Late Miocene unit exhibited hydraulic conductivity values ranging from almost zero to 0.8 m/d, averaging approximately 0.01 m/d.

## Discussion

**Figure 16:**
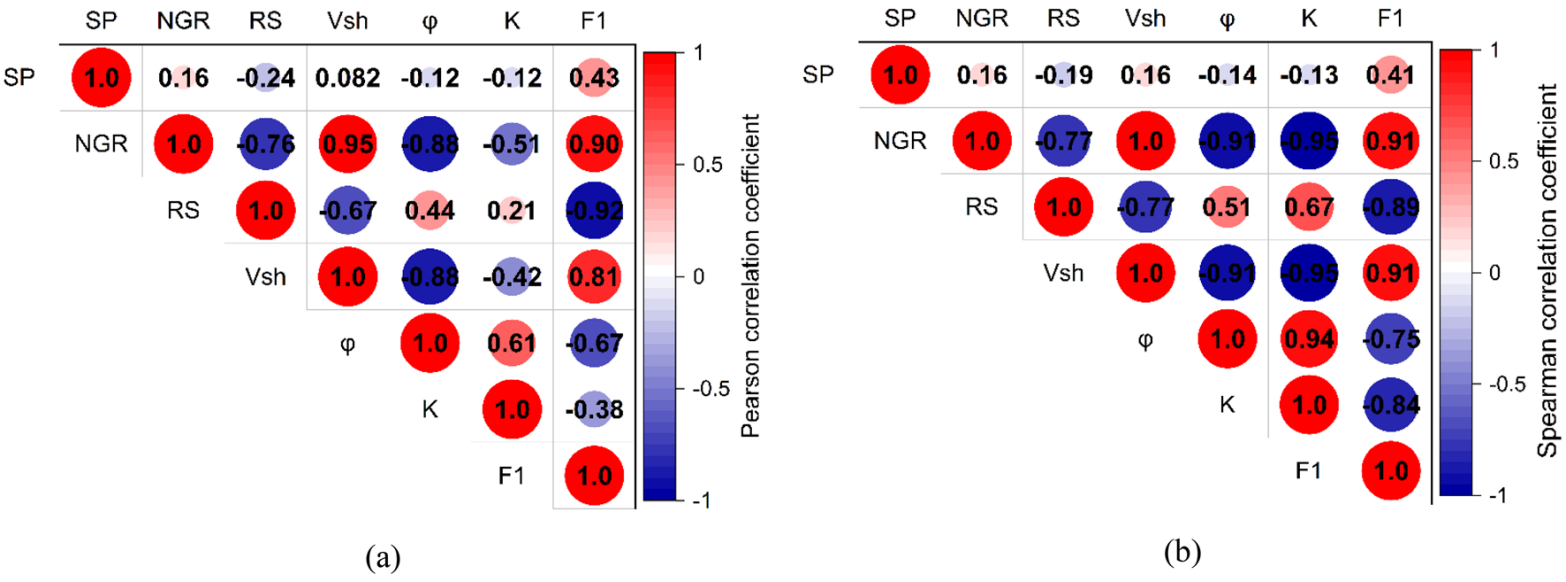
The correlation between the well logs, hydraulic parameters, and the extracted factor log using (**a**) Pearson and (**b**) Spearman correlation coefficients.

Factor analysis allowed the extraction of factor log that captured a significant portion of the data variance. Simple sensitivity analysis is conducted using Pearson and Spearman correlation coefficients (Fig. 16). These coefficients assist in understanding the relationship between well logs and the resulting factor logs and identifying which logs have the most significant impact on the outcome. The Pearson correlation coefficients, assuming linearity, displayed values of 0.43, 0.90, − 0.92, 0.81, − 0.67, and 0.38 between the extracted first factor and SP, NGR, RS, shale volume, effective porosity, and hydraulic conductivity, respectively (Fig. 16a). On the other hand, the Spearman rank correlation coefficients revealed stronger associations (0.41, 0.91, − 0.89, 0.91, − 0.75, and − 0.84). GR and RS logs exhibited higher correlations with the first factor because these logs are primarily sensitive to clay content, serving as indicators of lithological variation^70^. On the other hand, the SP log showed a lesser correlation with the extracted factor indicating its lower influence on the resulting factor log. This observation aligns with the initial hypothesis and underlines the dominant role of lithological characteristics in shaping the variability captured by the first-factor log.

Accordingly, the analysis of well-log data provided crucial implications for understanding the aquifer system in the study area. For instance, the variability in shale volume across the hydrostratigraphical units underscored the horizontal and vertical heterogeneity of subsurface geology^49,71^. The broad range of the estimated parameters depicted the heterogeneous nature of coarsening upward, alluvial, and Late Miocene units in which highly permeable materials coexisted within the less permeable zones^43^. The presence of low permeability shaly layers can act as barriers to flow, influencing the direction and velocity of groundwater movement. In contrast, the highly permeable sandy and gravely layers can facilitate rapid groundwater flow, potentially serving as potential aquifer zones^44^. The incised valley deposits, on the other hand, showed a more uniform distribution for the aquifer parameters with lower shale volume and higher effective porosity and hydraulic conductivity. The uniformity of this unit suggests a relative homogeneity, making it a potentially promising groundwater source^44,72^.

Factor analysis has proven to be a successful method for characterizing the main hydrostratigraphical units in the Debrecen area, considering the limited number of available well logs which is a notable limitation in recent investigations. Given this constraint, factor analysis emerged as a suitable method for the estimation of key petrophysical and hydrogeological parameters and facilitating the characterization of groundwater systems^38^. However, in the petroleum industry, where more comprehensive reservoir characterization is required, more sophisticated machine learning methods such as neural networks are commonly employed^73,74^. These methods offer high accuracy and flexibility in handling complex relationships between well-log data and target parameters^75^. However, they require larger datasets and computational resources for training and optimization. The factor analysis approach demonstrated a higher generalization ability in which the obtained practical equations can be safely used for estimating the characteristics of the clastic heterogeneous aquifers, especially within the Pannonian Basin. The shared geological history and lithological composition of these aquifers suggest favorable conditions for employing this factor analysis-based approach, However, slight fluctuations in the regression coefficient are expected due to the variation in saturation and degree of cementation^76^.

## Conclusion

The main aim of this research is to detect the vertical and horizontal distribution of the petrophysical and hydrogeological parameters within the main hydrostratigraphical units of the Quaternary system. This research demonstrated the potential of factor analysis in redefining the interpretation of well-log data. The conclusions of this research can be summarized as follows:

- The first factor extracted from the data matrix containing SP, NGR, and RS logs explained 81.7% of the data variance that showed a solid exponential relationship with the shale volume determined by the Larionov equation. This relation allowed the development of a universal equation that can be used independently for shale volume estimation. The shale volume estimated using this practical equation closely agrees with the deterministic approach.
- Based on the FA-based shale volume, the effective porosity is estimated and showed a close agreement with that of the deterministic approach. Moreover, a nonlinear relationship is obtained between the first scaled factor and the hydraulic conductivity. The FA-based hydraulic conductivity estimation revealed a significant correlation with the Csókás-based hydraulic conductivity, showing high variations within the hydrostratigraphical units. However, the distribution of hydraulic conductivity within the valley incision unit showed a more uniform pattern, making this unit a promising groundwater aquifer.
- The proposed methodology demonstrated potential for characterizing heterogeneous aquifer systems, and the findings can be directly applied to aquifers within the transboundary Pannonian Basin and other regions sharing similar geological and hydrogeological characteristics.

## Data Availability

The data that support the findings of this study are available from the Supervisory Authority for Regulatory Affairs (SARA), Hungary, but restrictions apply to the availability of these data, which were used under license for the current study, and so are not publicly available. Data are however available from the corresponding author (Musaab A. A. Mohammed) upon reasonable request and with permission of Supervisory Authority for Regulatory Affairs (SARA), Hungary.
